# Induced abortion incidence and safety in Burkina Faso in 2020: Results from a population-based survey using direct and social network-based estimation approaches

**DOI:** 10.1371/journal.pone.0278168

**Published:** 2022-11-30

**Authors:** Suzanne O. Bell, Georges Guiella, Meagan E. Byrne, Fiacre Bazie, Yentéma Onadja, Haley L. Thomas, Caroline Moreau

**Affiliations:** 1 Department of Population, Family and Reproductive Health, Johns Hopkins University Bloomberg School of Public Health, Baltimore, Maryland, United States of America; 2 Institut Supérieur des Sciences de la Population (ISSP), Université Joseph Ki-Zerbo, Ouagadougou, Burkina Faso; 3 Soins Primaires et Prévention, CESP Centre for Research in Epidemiology and Population Health, U1018, Inserm, Villejuif, France; Washington University in St. Louis, UNITED STATES

## Abstract

This study aims to estimate induced abortion incidence and safety in Burkina Faso using direct and indirect methods, overall and by women’s background characteristics. Data come from a nationally representative survey of reproductive aged women (n = 6,388). To address social desirability bias in abortion reporting, we asked about respondents’ closest female friends’ experience with abortion. The one-year abortion incidence in 2020 for respondents was 4.0 (95% CI 2.2–5.9) per 1,000 women aged 15–49 while the adjusted friend incidence was 22.9 (95% CI 15.8–30.0). Although not significant, abortion incidence was higher for adolescents, unmarried women, those with higher education, and those in urban areas among both respondents and their friends. Approximately nine out of ten abortions were unsafe (90% respondents, 95% friends), with respondent and friend findings suggesting higher risk of unsafe abortion among older women, less educated women, and women residing in rural areas. Despite recent increases in contraceptive use and continued legal restrictions, abortion remains common in Burkina Faso and is largely unsafe, with evidence of potential disparities.

## Background

In the West African country of Burkina Faso, induced abortion is legally permitted in cases of rape, incest, fetal impairment, or to save a woman’s life [1]. The country’s 1996 Penal Code states that any termination of a pregnancy outside of these defined circumstances results in imprisonment and heavy fines [2]. Nonetheless, abortion is common. Indirect estimates that adjust facility-based post-abortion complication rates (the Abortion Incidence Complications Methodology (AICM)) suggest there were approximately 25 induced abortions per 1,000 women of reproductive age (15–49) in 2008, corresponding to more than 87,000 abortions annually [3]. Investigators estimated that 43% of these abortions resulted in complications, less than two-thirds of which were treated in a health facility, with nearly 23,000 of these women hospitalized for abortion-related complications [3]. More recent findings from a global model of abortion rates suggest an annual rate of 30 (95% CI approximately 18–48) induced abortions per 1,000 women of reproductive age in 2015 to 2019 in Burkina Faso [4].

While these studies provide an overall assessment of the frequency and safety of abortion in Burkina Faso, we have limited information about who undergoes an abortion and who is most at risk of abortion-related morbidity and mortality due to unsafe abortion. Based on 2008 data, women living in urban parts of Burkina Faso, women with higher education, as well as women in their 20s, unmarried women, and those without children were more likely to have had an abortion [3], but the study provides no information on the determinants of unsafe abortion. A small qualitative study shows that lack of financial resources, education, and a less well connected social network result in delays in accessing services and social inequities in unsafe abortion [5]. Quantifying these disparities is paramount to improving maternal health, as unsafe abortion contributes to 10% of maternal deaths in the region [6]. Additionally, understanding who is most likely to use unsafe abortion to manage one’s fertility can highlight reproductive health disparities and inequities in the human right to safely decide whether and when to have a child.

This study of induced abortion incidence and safety in Burkina Faso is timely given the recent declines in the total fertility rate from 6.0 children per woman in 2010 [7] to 5.2 in 2017 [8] and accelerated uptake of modern contraceptive use from 14% in 2010 [7] to 29% in 2020 [9]. At the same time, the abortion landscape has shifted, with increased availability and use of misoprostol for self-managed abortion [10]. Despite these recent changes, 26% of recent pregnancies were unintended, postabortion care remains inadequate, and the maternal mortality rate remains high at 320 deaths per 100,000 live births [9, 11–13]. With high rates of unintended pregnancy, limited access to safe abortion and postabortion services, and significant costs to women and their households [14], updated estimates of induced abortion incidence and safety are needed to guide programmatic and policy efforts to better meet women’s reproductive needs in Burkina Faso.

Measurement of induced abortion incidence and safety has also evolved since in recent years as investigators have begun using existing indirect methodologies to address social desirability bias and better capture clandestine abortions that are becoming safer with the diffusion of abortion pills outside of the formal healthcare system. Emerging from early work that relied on respondents’ reports of their siblings’ sensitive behaviors or events [15, 16], Rossier *et al*. (2006) first adapted this indirect approach to estimate abortion incidence in Ouagadougou, Burkina Faso by asking respondents about all their close friends’ experience with abortion; they referred to this method as the Anonymous Third Party Reporting (ATPR) [17]. Other investigators subsequently modified this method, using different friend definitions that either require mutual disclosure of sensitive information (confidante method, similar to the ATPR friend definition) [18] or just a close female friend (best friend method) [19], and different fixed numbers of friends (one, two, or three instead of all) [19–21] in an effort to improve method performance. These methods broadly assume that 1) women have close friends who are similar in the aggregate with regard to socioeconomic and reproductive characteristics (i.e., no selection bias in the surrogate sample of female friends), 2) women share their abortion experience with their close friends (i.e., no transmission bias); and 3) reporting of abortion is improved when talking about friends versus self (i.e., reduced social desirability bias). Studies using social network-based methods to estimate abortion incidence have generally produced higher estimates of abortion incidence, however, results have been mixed in relation to the extent of assumption violation and the success of subsequent adjustments for observed biases [20, 22–26]. While social network-based method assumption violations may bias estimates of abortion incidence, much of the current global estimates rely heavily on AICM estimates, which may be biased in other ways as they rely on untestable key informant assessments of the extent of clandestine abortions that don’t lead to hospitalization to adjust facility-based postabortion care rates. Thus, we believe refined social network-based methodologies–that attempt to adjust for assumption violations–are warranted to complement AICM estimates, providing an opportunity to triangulate results using different data sources (facility-based in the case of the AICM and population-based in the case of social network-based approaches) with different potential biases.

The current study seeks to provide updated national estimates of induced abortion incidence and safety in Burkina Faso using direct questions and the best friend approach, whereby respondents report on their closest female friend’s experience with abortion [27]. We anticipated that this social network method would perform well in Burkina Faso as the prior ATPR estimates in Ouagadougou were congruent with hospital data in terms of abortion rate levels and age patterns [17] and abortions may be more visible between friends in a context where women rely on their social network to access abortion given legal restrictions [28]. We estimate the one-year abortion incidence and abortion safety overall and by background characteristics after evaluating and adjusting for assumption violations.

## Methods

### Data

Data for this study come from the Performance Monitoring for Action (PMA) project [29]. PMA conducts annual population-based surveys of households and reproductive age women enrolled in a panel study in eight countries. In Burkina Faso, the household survey design utilized an urban/rural stratified two-stage clustered sampling approach with probability proportional to size selection of clusters based on the population size within each stratum. The PMA sample size was calculated to estimate the modern contraceptive prevalence rate within a three-percentage point margin of error at the national level and within a five-percentage point margin of error within urban and rural strata. PMA received local permission before beginning data collection within a selected cluster, sharing letters documenting federal and ethical review board approval for the study. In each cluster, interviewers mapped and listed all households (administrative units comprised of approximately 200 households) and Supervisors randomly selected 35 households. All women aged 15–49 years from selected households were invited to participate. Interviewers made up to three attempts to interview a household respondent and each eligible women identified in the household roster. The first round of data collection was conducted from December 2019 through February 2020. The final sample for the first round included 5,696 completed household surveys (98.8% response rate) and 6,590 completed female surveys (95.8% response rate). We calculated response rates as the number of completed surveys among eligible households and among reproductive aged women.

Data for the current study come from the second round of data collection in Burkina Faso, which occurred from December 2020 through March 2021. We included all panel women who completed a second round of data collection who still resided in sample clusters (n = 5,310, 81% follow-up). To account for attrition, PMA randomly selected replacement households equal to the number of households lost to follow-up from within clusters that had more than 10% loss to follow-up to produce cross-sectional, nationally representative estimates of reproductive health indicators. The replacement households were selected from an updated cluster sampling frame, which interviewers created via a new mapping and listing of all households prior to Round 2 data collection. With the addition of the replacement households and associated women (n = 1,403), the round two data included a total of 5,522 households (97.9% response rate) and 6,388 women (93.5% response rate) who provided verbal informed consent to participate and completed the survey. We constructed survey-design weights using the inverse of the cluster and household selection probabilities and further adjusted the weights for non-response at the household and individual level within the cluster.

Local trained female interviewers implemented the surveys face-to-face, soliciting information on women’s socioeconomic characteristics, reproductive history, and knowledge and use of contraception, as well as an abortion module described in more detail below. The female questionnaire was developed in French and administered in French or a local language using translations decided upon by interviewers and the research team familiar with each language during the training. The Institutional Review Board at the Johns Hopkins University Bloomberg School of Public Health and the Comité d’Ethique pour la Recherche en Santé/Ministère de la Santé et de l’Hygiène Publique et Ministère de l’Enseignement Supérieur, de la Recherche Scientifique et de l’Innovation in Burkina Faso provided ethical approval for the study.

### Measures

The abortion module, which builds off prior PMA abortion work in other countries [20, 22–24], included questions about respondent’s and their closest female friend’s experience with abortion. Friends were defined as being between the ages of 15 and 49 and living in Burkina Faso. For a separate methodological analysis, half of respondents were randomly asked to report on their closest female friend with whom they mutually share sensitive information while the other half reported on their closest female friend with no other specification [30]. Given inconclusive findings regarding which definition produced more accurate results, we combined the friend definitions for the current analysis. Respondents provided demographic information about their closest female friend, as well as information about their friend’s use of contraception and experience with abortion. The last section of the module asked respondents about their own abortion experience. Abortion was described using two terminologies in separate questions: doing something to “end a pregnancy” and then “bringing back a late period”. For those who reported doing something to bring back a late period, we asked whether the motivation was because they were worried they were pregnant as women may regulate their periods for other reasons [31]. Further details on the PMA abortion terminology are provided elsewhere [23, 31, 32]. After each of these questions, interviewers asked whether it was intentional (or occurred naturally) and whether the actions were successful. For the most recent intentional, successfully ended pregnancy or period regulation (henceforth referred to as “abortion”) that occurred in the last 10 years, interviewers collected information regarding the year, method(s) and source(s) used. For friend abortions, we included those that the respondent reported with certainty (“Yes, I am certain”) or less certainty (“Yes, I think so”).

We considered the following sociodemographic and reproductive health information for respondents and their friends: age, education, current marital status, residence, parity (nulliparous, parous), current use of any contraception, and current use of long-acting reversable contraception (LARC) specifically. We also considered wealth tertiles for respondents, which we derived from a continuous wealth measure using principal components analysis from information on household assets, water, sanitation, and building materials following a similar method to that employed by the Demographic and Health Surveys; we were unable to collect this information for friends.

We grouped abortion methods into five categories, including surgery, medication abortion pills (misoprostol with or without mifepristone), other identified pills (e.g., contraceptive pills, antimalarial pills, antibiotics), unknown pill type, injection, and traditional or other methods (e.g., herbs, home remedies like bleach). We grouped abortion sources into four categories, including public facilities (national hospital center, regional hospital center, health and social services center, family planning clinic, public medical center, public medical center with surgical unit, public mobile outreach clinic), private facilities (private hospitals/clinics, private practices, private doctors, maternities, other private providers), pharmacy, and traditional/other (fieldworkers/community health volunteers, health agents, shops/markets, religious organizations, community events, traditional healers, friends/relatives, street vendors, house, and other). We separated public and private facilities to provide insight into which sector women are more likely to use for abortion and postabortion care, if any, given these facilities operate differently and have different managing authorities.

To determine abortion safety, we constructed a three-category variable incorporating information on all methods and sources used. We defined the categories as 1) safe, involving a recommended method (i.e., surgery or medication abortion pills) in a healthcare facility; 2) less safe, involving a non-recommended method in a healthcare facility or a recommended method from outside a healthcare facility, and 3) least safe, involving a non-recommended method outside of a health facility. These categories roughly align with the World Health Organization’s (WHO’s) abortion safety definitions used for global estimates if we assume healthcare facilities meet the minimum medical standards and appropriately trained providers criteria for safe abortion [33]. To address the 2022 WHO safe abortion guidelines that now include self-managed medication abortion [34], we constructed an alternative three-category abortion safety variable which reclassified all medication abortions pills–regardless of source–as safe. We then dichotomized this second version by combining less safe and least safe into a single unsafe category. For sensitivity analyses we also reclassified unknown pills from facilities as medication abortion pills, and all unknown pills as medication abortion pills to examine the impact of potential safe abortion misclassification.

### Analyses

We began by exploring potential biases in the friend data. To assess potential selection bias of the friend surrogate sample (assumption 1) and transmission bias (assumption 2), we first compared the characteristics of respondents who reported having 0 or 1 or more friends by sociodemographic (age, education, marital status, residence, wealth tertile) and reproductive (parity, any contraceptive use, LARC use) characteristics. We then compared these same sociodemographic and reproductive characteristics (except wealth) among respondents and friends to determine the representativeness of the friend surrogate sample. We used design-based F-tests to assess statistical significance of observed differences. To further evaluate potential transmission bias, we determined the percent of respondents who shared their own abortion experience with their friend. To examine whether social desirability was reduced (assumption 3), we compared respondent and unadjusted friend abortion rates.

Next, we adjusted the friend surrogate sample for violations of social network method assumptions related to selection bias and transmission bias. To account for the respondents who reported having no female friends–which potentially introduces selection and transmission bias and violates assumptions 1 and 2 –we incorporated this sub-population of respondents into the surrogate sample as the surrogate sample is essentially missing women who have no friends. Given we know self-reported abortion data underestimates abortion incidence, we then used a Poisson model to predict the likelihood of these “missing” friends having had an abortion in the prior year by regressing the respondents’ socioeconomic and reproductive characteristics on the observed friend abortion incidence data, including whether the respondent reported having an abortion in the prior year. To further improve the representativeness of the surrogate sample, we constructed post-stratification weights for the surrogate sample to replicate the sociodemographic distribution of the respondent sample, which is nationally representative of women aged 15 to 49. We used these adjusted friend data to estimate abortion incidence. We used design-based F-tests to evaluate whether respondent and adjusted friend characteristics were statistically significantly different. To adjust for transmission bias, we multiplied friend abortion rates by the inverse probability of respondents sharing information about their own abortion with their closest friend [18, 21, 25, 26]. Given we observed higher sharing for pregnancy removal than period regulation, we accounted for the distribution of the abortion types and respective sharing in calculating the transmission bias adjustment factor. This adjustment assumes the level of abortion sharing is similar in both directions (respondents to friends and friends to respondents), on average. While we were unable to identify an appropriate statistical test for comparing the adjusted friend abortion incidences to the respondent incidences given the *post hoc* transmission bias adjustment, we used the non-overlap of confidence intervals to determine whether differences were significant.

We only collected data on year of abortion, thus we included those reported in 2020 and early 2021, dividing by the average number of years between January 2020 up until the date of the interview (1.06 years) to account for potential misplacement and calculate an annual rate. We then multiplied the estimate (and standard errors) by 1,000 to produce an annual rate of induced abortions per 1,000 women aged 15 to 49. We present these estimates overall and by background characteristics separately for respondents and their friends. While we assume the respondent abortion rates will be underestimated, we nonetheless include the respondent results as a comparison to the friend estimates to reveal the extent of underreporting and to provide additional evidence regarding the likely patterns of abortion incidence in this context.

With regard to abortion safety, we used the aforementioned three-category safety variables relying on abortion method and source information. To account for the missing friends’ abortions and any associated selection bias, we included the abortion details of respondents who reported having 0 friends in the surrogate sample of friends’ abortions, similar to our adjustment in the incidence analysis. We examined the distribution of abortion safety for respondents and friends overall. We tested for differences in the percent of abortions that were unsafe by background characteristics using design-based F tests. Lastly, given the large percentage of women who reported they or their friend used pills of an unknown type to terminate, we conducted two sensitivity analyses to evaluate the impact on safety estimates: 1) assuming all unknown pills were medication abortion pills, and 2) assuming only unknown pills from facilities were medication abortion pills.

We conducted all analyses in Stata version 15.1 [35]. Given the complex sampling design, we applied survey-design weights and calculate standard errors using the Taylor linearization to account for clustering.

## Results

### Sample characteristics

A total of 6,388 women of reproductive age completed the survey, 5,042 (78.9%) of whom reported having at least one close female friend. Women reported 1.5 close friends on average. S1 Table presents the characteristics of respondents overall and by whether they reported having any close female friends of reproductive age. Results indicate that, compared to women who had 0 close friends, women who had at least one friend were significantly younger (21.3% age 15–19 versus 17.5%) and more educated (25.9% with secondary or tertiary education versus 18.1%). Respondent marital status, household religion, wealth tertile, residence, parity, current contraceptive use, current LARC use, and the induced abortion rate were all similar by whether they reported having any close friends.

Table 1 shows the demographic characteristics of the respondents and the unadjusted and adjusted characteristics of their close female friends. Most respondents were in their 20s (33.5%), had no formal schooling (57.5%), resided in rural areas (77.8%), were married (75.4%), and had at least one child (76.8%). One-third (32.3%) of respondents reported currently using a contraceptive method while 13.9% reported using a LARC method specifically. The distribution of the close friend sample differed minimally but statically significantly from the respondent sample by education level, residence, and parity even after adjusting close friend data to account for respondents who did not report a close friend (all p<0.01) (Table 1). Specifically, close friends were somewhat more educated (24.3% of respondents with secondary or tertiary education, 25.9% of friends), were more likely to reside in urban areas (22.2% for respondents, 25.7% for friends), and less likely to be nulliparous (23.2% for respondents, 25.1% for friends). Respondents and friends had similar age, marital status, and current contraceptive use (including LARC use specifically) after adjustment (Table 1).

**Table 1:** Characteristics of female respondents aged 15 to 49 and their closest female friends age 15 to 49 in Burkina Faso*.

	Respondent		Unadjusted friend		Adjusted friend**	
	%	N	%	N	%	N
Age						
15–19	20.6	1350	20.2	983	20.4	1250
20–29	33.5	2232	34.5	1692	34.8	2127
30–39	28.0	1750	27.7	1428	27.2	1837
40–49	17.9	1055	17.6	884	17.7	1174
Education						
Never	57.5	2682	**57.1**	2104	**57.7**	2749
Primary	18.2	1299	**14.3**	809	**16.5**	1124
Secondary	22.5	2121	**26.2**	1829	**23.8**	2182
Tertiary	1.8	284	**2.4**	283	**2.1**	331
Currently married						
No	24.6	2123	**26.3**	1673	25.9	2063
Yes	75.4	4265	**73.7**	3369	74.1	4325
Wealth tertile						
Poorest	34.2	1183	--	--	--	--
Middle wealth	31.8	1318	--	--	--	--
Wealthiest	34.0	3887	--	--	--	--
Residence						
Rural	77.8	2615	**73.4**	2045	**74.3**	2597
Urban	22.2	3773	**26.6**	2997	**25.7**	3791
Parity						
0	23.2	1875	**26.9**	1610	**25.1**	1939
1+	76.8	4510	**73.1**	3429	**74.9**	4446
Currently using contraception						
No	67.7	3998	67.5	3253	68.2	4170
Yes	32.3	2390	32.5	1789	31.8	2218
Currently using LARC						
No	86.1	5490	84.5	4213	85.0	5384
Yes	13.9	898	15.5	829	15.0	1004
Total	100.0	6388	100.0	5042	100.0	6388

*Estimates weighted, Ns unweighted; bold indicates p-value for design-based F-test (reference respondents) less than 0.05

**Estimates include respondent characteristics in place of "missing" confidantes; post-stratification weights applied

### Induced abortion incidence estimates

The one-year induced abortion incidence rate in 2020 among respondents was 4.0 (95% CI: 2.2–5.9) per 1,000 women aged 15–49 years while the unadjusted friend rate was 13.6 (95% CI: 8.2–19.0) before accounting for selection and transmission bias. Overall, 61.8% of the 137 respondents who reported having had an abortion shared this information with their closest female friend, with higher sharing for ending a pregnancy (77.6%) than period regulation (54.6%) (estimates not shown). Accounting for the distribution of abortions reported as ending a pregnancy (35.6%) versus period regulation (65.5%), the inverse probability of sharing produced a transmission bias adjustment factor of 1.64. The friend incidence increased substantially to 22.3 (95% CI: 13.5–31.2) when adjusting for transmission bias (estimate not shown) but was minimally affected when further adjusting for selection bias; the final adjusted friend estimate was 22.9 (95% CI: 15.8–30.0) per 1,000 (S2 Table).

**Fig 1:**
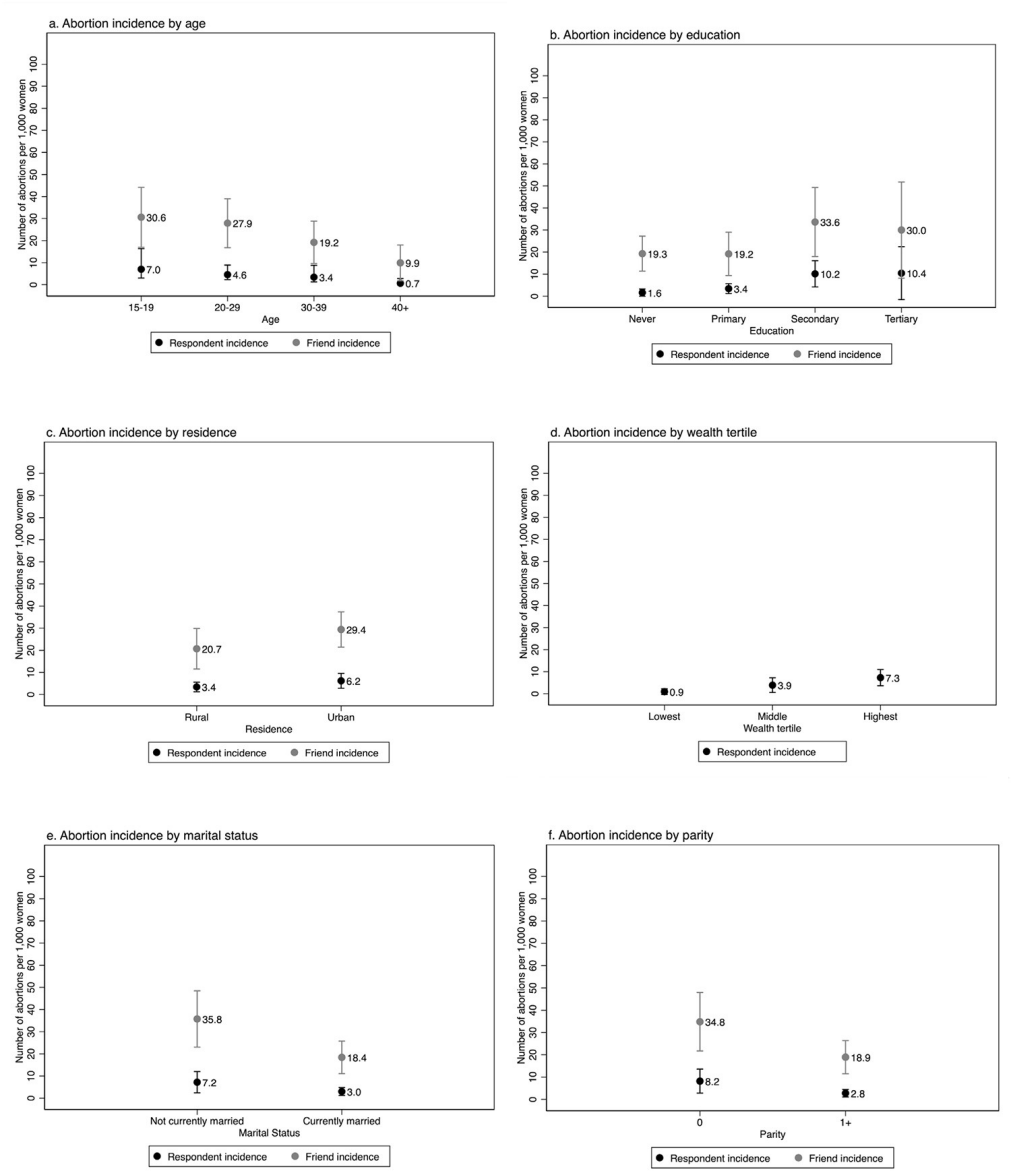
One-year annual incidence of induced abortion per 1,000 women aged 15–49 for respondents and friends in Burkina Faso by background characteristics, 2020.

Friend induced abortion incidence estimates were significantly higher than those of respondents across most characteristics, although they followed similar patterns by background characteristics (Fig 1 and S2 Table). Abortion incidence varied by age, parity, and marital status, with the highest incidence among teenagers 15–19 years (7.0 for respondents, 30.6 for friends), unmarried women (7.2 for respondents, 35.8 for friends) and women with no children (8.2 for respondents, 34.8 for friends). Incidence also varied by residence, education, and wealth, with the highest incidence among women residing in urban areas (6.2 for respondents, 29.4 for friends), women who attended secondary school or tertiary school (10.2 and 10.4, respectively for respondents, 33.6 and 30.0, respectively for friends) and respondents in the highest wealth tertile (7.3); we could not determine wealth for friends.

### Induced abortion methods and sources

We have data on respondent and friend induced abortion characteristics for a total of 137 and 286 abortions occurring in the past 10 years, respectively (310 friend abortions when adjusted to include missing friends, i.e., respondents who reported having 0 friends). Respondents mostly relied on traditional or other means (32.0%), followed by non-recommended pills (i.e., contraceptive, antimalarial, and antibiotic pills) (28.4%), unknown pill types (24.8%), injections (9.6%), medication abortion pills (7.3%), and surgery (3.7%) (Table 2). Unknown pills, traditional/“other” methods, and non-recommended pills were also most commonly used by friends (33.6%%, 29.3%, and 22.9%, respectively), followed by injection (8.6%), surgery (2.8%), and medication abortion pills (2.2%). Some respondents were not able to report which methods they (1.1%) or their friend (8.2%) used. The percentage who used medication abortion pills, unknown pill types, or other unknown methods differed significantly between respondents and friends. Results for abortion source did not differ significantly between respondents and friends (Table 2). Public facilities (43.5% for respondents and 47.0% for friends) and other non-clinical sources (44.6% and 36.9%) were the two most commonly reported sources. Private facilities (9.2% of respondents and 5.5% of friends) and pharmacies (8.2% of respondents and 8.1% of friends) were less commonly used while source was unknown for 6.6% of friends.

**Table 2:** Details of most recent reported induced abortion among female respondents aged 15 to 49 and their closest female friends aged 15 to 49 in Burkina Faso*.

	Respondent		Adjusted friend**	
	%	N	%	N
All methods used (multiple select)				
Surgery	3.7	9	2.8	13
Mifepristone/misoprostol pills	7.3	9	**2.2**	11
Other pills (identified)	28.4	47	22.9	72
Unknown pill type	24.8	28	**33.6**	88
Injection	9.6	15	8.8	21
Traditional/other methods	32.0	38	29.3	100
Do not know/No response	1.1	3	**8.2**	35
All sources used (multiple select)				
Public facility	43.5	55	47.0	119
Private facility	9.2	21	5.5	35
Pharmacy	8.2	20	8.1	31
Other non-clinical	44.6	49	36.9	124
Do not know/No response	0.0	0	6.2	24

*Estimates weighted, Ns unweighted; bold indicates p-value for design-based F-test (reference respondents) less than 0.05

**Estimates include respondent abortion characteristics in place of "missing" friends if respondent reported 0 friends and an abortion; post-stratification weights applied

### Abortion safety

Table 3 presents the distribution of abortion safety overall for respondents and close friends. Based on the initial WHO abortion safety definition, 8.9% of respondent abortions were safe, 45.3% were less safe, and 45.0% were least safe; the corresponding estimates for friends were 3.8%, 46.8% and 49.4%. While not statistically significantly different, the difference in these distributions was driven by the lower percentage of friends using medication abortion pills and the higher percentage of friends using unknown pills or an unknown method. When reclassifying all self-managed medication abortions as safe in line with the 2022 WHO guidelines, safe abortion rose only slightly to 10.1% for respondent and 4.8% for friends, while less safe abortions dropped to 44.1% for respondents and 45.8% for friends (Table 3). In sensitivity analyses where we categorized all unknown pills as medication abortion, the percentage of safe abortions rose to 34.7% and 38.2% for respondents and friends, respectively (estimates not shown). If we only recategorized unknown pills provided by health facilities as medication abortion, the percentage of safe abortions rose only to 20.6% for respondents and 25.8% for friends (estimates not shown).

**Table 3:** Safety of most recent reported induced abortion among female respondents aged 15 to 49 and their closest female friends aged 15 to 49 in Burkina Faso*.

	Respondent		Adjusted friend**	
	%	N	%	N
Current WHO safety measurement***				
Safe	8.9	13	3.8	16
Less safe	45.3	67	46.8	128
Least safe	45.8	57	49.4	166
With new self-managed MA reflected****				
Safe	10.1	16	4.8	22
Less safe	44.1	64	45.5	122
Least safe	45.8	57	49.4	166
Total	100.0	137	100.0	310

*Bolding indicates statistically significantly different at the p<0.05 level (reference respondent)

**Adjusted friend data includes respondent abortion details for respondents who reported having no friends

***Surgery and medication abortion from clinical source = safe

****Surgery from facility and medication from any source = safe

**Fig 2:**
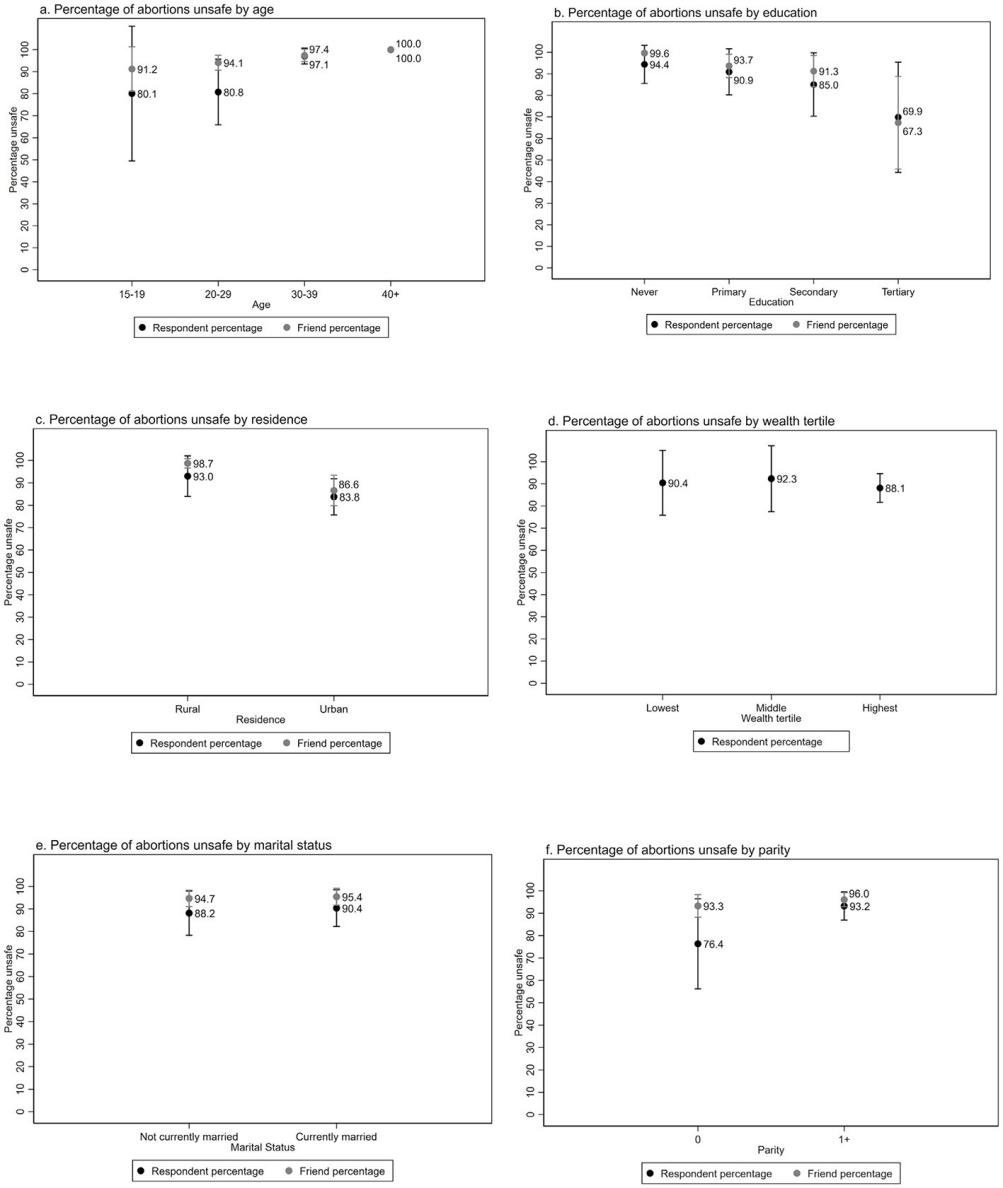
Percent of induced abortions that were unsafe among female respondents aged 15 to 49 and their closest female friends aged 15 to 49 in Burkina Faso by background characteristics.

Using the abortion safety definition that is most aligned with current WHO safe abortion guidelines (i.e., self-managed medication abortion categorized as safe), we present the percent of abortions considered unsafe (less safe and least safe combined) for respondents and friends by background characteristics in Fig 2 (and S3 Table). Overall, 89.9% of respondents’ abortions and 95.2% of friend abortions were unsafe (S3 Table). For both respondents and friends, the likelihood of having an unsafe abortion was lowest for adolescents (80.1% for respondents, 91.2% for friends) and highest for women aged 40–49 (100.0% for both respondents and friends). By education, we observed the greatest risk of unsafe abortion for women with no education (94.4% for respondents, 99.6% for friends) and the lowest risk for those with tertiary education (69.9% for respondents, 67.3% for friends). There were no meaningful differences by marital status or wealth, while women in rural areas were more likely to have had an unsafe abortion (93.0% for respondents, 98.7% for friends) than women in urban areas (83.8% for respondents, 86.6% for friends). Respondents with no children were significantly less like to have had an unsafe abortion than those with any children (76.4% compared to 93.2%); the pattern was less pronounced for friends at 93.3% and 95.8%, respectively. Respondent differences in unsafe abortion by parity were statistically significant while respondent unsafe abortion by age was borderline significant (p-value = 0.09). Friend abortion safety estimates were significantly different by education and residence (p<0.01).

## Discussion

The present study provides the most recent non-model-based estimates of induced abortion incidence and safety in Burkina Faso since 2008 [3] using nationally representative population-based data. At 22.9 (95% CI 15.8–30.0) abortions per 1,000 women of reproductive age in 2020, our friend estimate is similar to the 2008 estimate of 25 (no confidence interval provided) per 1,000 derived from adjusted postabortion care rates [3] and lower than the modeled estimate of 30 (95% CI 18–48) for 2015 to 2019 [4], although confidence intervals largely overlap. Triangulation across these three different methodologies, each with different limitations, suggests the induced abortion rate for Burkina Faso is likely between 23 and 30. Thus, the use of the best friend method in population-based surveys is a reasonable strategy to estimate abortion incidence rates and gain more insight on the social determinants and conditions of abortion care in this setting.

Our results offer greater understanding of the circumstances of induced abortion in Burkina Faso, which depend on the reproductive life course and socio-economic context of the woman. Induced abortion is more common at the start of women’s reproductive careers, among young nulliparous and unmarried women, reflecting social stigma attached to premarital childbearing. Induced abortion is also more common among more affluent women, more educated, wealthier, and urban women, signaling an acceleration of the demographic transition in this population that is also more likely to use abortion to achieve lower desired fertility. These results are consistent with those reported in two other West African countries [3, 23, 24] and align with prior findings from Burkina Faso in 2008 [3]. However, these patterns may not reflect underlying abortion demand as structural and financial constraints likely limit women’s ability to access abortion care. Results nonetheless highlight the continued need to invest in family planning programs across the country to meet the changing fertility preferences of women in Burkina Faso.

Investments in programs to reduce unsafe abortion and its negative sequelae are of particular significance given that nine out of ten induced abortions in Burkina Faso are unsafe. Our results are similar to the WHO’s model estimates of 85% (90% CI 76–90) unsafe abortion for the West African region, largely based on facility-based input data [33] but higher than recent population-based estimates from Nigeria (71%) and Cote d’Ivoire (67%) using a similar methodology as our study [23, 24]. Disparities in unsafe abortions are also noted within country, although the statistical power of our analysis was limited due to the small sample size of abortions. The social patterning of unsafe abortion in Burkina Faso by education, wealth and residence mirror findings in Nigeria and Cote d’Ivoire [23, 24], suggesting women with less socio-economic or geographic resources are less likely to access safe abortion services. Research has shown these same groups are less likely to seek postabortion care services when experiencing complications, contributing to a double burden of disease by augmenting their risk of maternal morbidity and mortality [36–38]. Barriers to postabortion care include stigma [39] and cost, as described in a recent study indicating that women often paid more than quadruple the price limit on postabortion care services (US$7) to treat abortion complications [14]. In addition, many facilities are not prepared to provide quality postabortion care [13]. Harm reduction strategies aimed at increasing access to self-managed medication abortion could also significantly reduce unsafe abortion. This would require increased availability and knowledge of medication abortion pills, complemented by efforts to improve postabortion care availability and quality to support those who require treatment for incomplete abortion or complications.

Our findings need to be interpretated with several limitations in mind. While we anticipate the self-reported abortion rates are substantial underestimates, friend estimates are not inherently more accurate. The best friend methodology relies on several assumptions [26]. In the absence of a validated data source on abortion, it is not possible to evaluate the extent to which the friend incidence estimates are accurate. Instead, we rely on a series of assumption assessments and adjustments to reduce potential bias in friend estimates, including adjusting for transmission bias, which increases our initial estimates by 64%. This adjustment assumes that respondents who report an abortion have similar transmission patterns (61% based on our results) as the friends in the surrogate sample. However, if respondents who do not disclose their abortion in a survey are less likely to share their experience with a friend, we would underestimate the transmission bias, leading to underestimation of friend abortion rates.

Another assumption of this social network-based methodology is that the surrogate sample is representative of the general population of women of reproductive age. One concern is the possibility that some popular women are represented more than once in the surrogate sample, leading to potential bias if popular women have different abortion rates. However, the likelihood of double counting friends is low since the sample includes 35 out of approximately 200 households from each geographic cluster and respondents could report on friends living anywhere in the country. Additionally, there is a possibility of friend selection bias if the reported friends shared their abortion experience more than the average person who has had an abortion; this would bias our friend abortion incidence estimates upward. We were unable to explore this assumption directly, though complementary work examining the impact of the friend definition on induced abortion incidence estimates in Burkina Faso suggests this may be a concern when specifying a friend with whom the respondent shares sensitive information versus not [30]. Half of the friend surrogate data in our study did not include sharing of sensitive information criteria in the friend definition, reducing the aforementioned bias. The issue of “missing” friends with no social network (corresponding to the 21% of respondents with no friends) is another concern, which can affect abortion rates if abortion rates differ by whether a woman has a social network or not. However, abortion rates among respondents who reported having 0 versus 1 or more friends were not significantly different (4.9 versus 4.2) and is accounted for in the adjusted estimates. Ultimately, the magnitude of differences between the friend and adjusted surrogate samples were small (less than 2 percentage points, except for residence) and showed similar patterns of contraceptive use, a validation approach suggested by others [26]. The adjustment of abortion incidence accounting for surrogate sample selection bias was minimal.

While underestimation of transmission bias could lead to an underestimation of the friend abortion rate, further underestimation could be due to the exclusion of repeat abortions in the same year. On the other hand, our categorization of period regulations when women were worried they were pregnant as abortions may have led to the inclusion of non-abortions in the final estimates. In the end, the induced abortion incidence patterns are similar for respondents and friends, providing support to the notion that these patterns reflect reality, but it could alternatively be the case that the respondent and friend data are biased in similar ways. However, many of these patterns are consistent with findings from other low- and middle-resource settings [23, 24, 40–42].

Our results provide an understanding of the abortion safety landscape in Burkina Faso, and are in line with the prevalence of unsafe abortions published by WHO using a different methodology [33]. But several limitations call for cautious interpretation of our findings. First, our sample of respondents reporting abortions was small (n = 137), limiting our precision with regard to induced abortion safety estimates and our ability to identify clear and significant patterns across sociodemographic characteristics. Second, to the extent that respondent abortion reporting (or knowledge of and reporting on their friend’s abortion) is differential based on safety, our safety estimates would be biased. However, triangulation with other induced abortion safety estimates for the region obtained via a different methodology and similarity between respondent and friend estimates suggests our findings are likely close to the true value. Third, several measurement limitations likely contribute to safety misclassification. Some facility-based surgical procedures are wrongly classified as safe if they are performed by untrained providers or follow obsolete procedures (curettage). This information cannot be captured from women in a population-based survey and would need facility-based information on quality of abortion care to adjust the estimates. Conversely, facility-based injections, reported by about 10% of women, were classified as less safe as they involve non-recommended methods, but they could have been administered for cervical ripening before surgical abortion. Only one woman reported having both an injection and a surgery, thus we believe this misclassification was minimal. Self-managed abortions are also difficult to classify as they fall along a continuum of safety based on the pills, regimen, accompanying information, and access to postabortion services in case of complications. In our study, only 1% of abortions involved self-managed medication abortion, but self-managed medication abortion could be more common if some of the unknown pills were medication abortion pills. Our sensitivity analysis suggests up to 35% of abortions would be considered safe if all unknown pills were in fact medication abortion pills. We find it unlikely a majority of these pills were medication abortion pills given low knowledge of this method [43]. In addition, medication abortion regimens and information were not assessed in our study, falling short of the self-managed abortion safety definition recommended by the WHO [34].

Despite these limitations our study has several strengths. Data are from a large, nationally representative sample, which affords greater visibility of self-managed abortion and better captures women obtaining unsafe abortions. Our use of a social network-based indirect method allowed us to estimate a more valid 1-year induced abortion incidence than self-report by mitigating social desirability bias. We also employed several adjustments to counteract observed biases. In addition, we collected data on background characteristics of both respondents and their closest friends, which allowed us to analyze incidence and safety estimates by characteristics using individual-level data to better triangulate estimates and patterns. This provides insight into who is most likely to have an abortion as well as who is most vulnerable to unsafe abortion, allowing for more specific public health measures to be put in place to mitigate negative health outcomes for those populations. To further refine our understanding of the continuum of abortion safety, future research should integrate quality of care and social safety measures as well as triangulate population-based and facility-based information to fully reflect the WHO’s recommendations for abortion safety. Such efforts would help identify priorities for interventions, including quality of services, addressing social stigma, and information campaigns to enable safe self-managed abortions.

## Conclusion

Using the best friend methodology, we estimate that the induced abortion incidence in Burkina Faso has remained relatively stable since 2008, with an incidence of 23 abortion per 1,000 women of reproductive age in 2020 [3]. Our application of the best friend approach provides a complementary perspective from facility-based postabortion care derived measures and modeling approaches [3, 4] to understand the changing landscape of abortion in this region of the world. Approximately nine out of ten induced abortions in the country are unsafe, and beyond safety, many are likely to involve poor quality care. For those who self-manage their medication abortion, they cannot rely on an enabling environment to achieve self-care standards. Further expansion of legal indications for safe abortion and greater access to quality and affordable comprehensive reproductive health services are needed to reduce the health burden of unsafe abortion, which remains a significant cause of maternal mortality in Burkina Faso.

## Supporting information

S1 Table Characteristics of female respondents aged 15 to 49 overall and by whether they report having at least one close female friend in Burkina Faso. (PDF)

S2 Table Induced abortion incidence (per 1,000) among female respondents aged 15 to 49 and their closest female friends aged 15 to 49 in Burkina Faso by background characteristics. (PDF)

S3 Table Percent of induced abortions that were unsafe among female respondents aged 15 to 49 and their closest female friends aged 15 to 49 in Burkina Faso by background characteristics. (PDF)
