# Supplementary material for: Induced abortion incidence and safety in Burkina Faso in 2020: Results from a population-based survey using direct and social network-based estimation approaches
Source: PLoS One. 2022 Nov 30;17(11):e0278168. doi: 10.1371/journal.pone.0278168 (PMC9710743; doi:10.1371/journal.pone.0278168)
Supplement: S1 Table — (PDF) [file pone.0278168.s001.pdf]

S1 Table. Characteristics of female respondents aged 15 to 49 overall and by whether they reported having at least one close female friend in Burkina Faso*

	All respondents		0 Friends		≥ 1 Confidante	
	%	N	%	N	%	N
Age						
15-19	20.6	1350	**17.5**	249	**21.3**	1088
20-29	33.5	2232	**31.0**	403	**34.1**	1815
30-39	28.0	1750	**29.5**	380	**27.7**	1361
40-49	17.9	1055	**22.1**	269	**16.8**	777
Education						
Never	57.5	2682	**62.8**	615	**56.0**	2042
Primary	18.2	1299	**19.1**	299	**18.1**	993
Secondary	22.5	2121	**16.6**	339	**24.0**	1771
Tertiary	1.8	284	**1.5**	46	**1.9**	236
Currently married						
No	24.6	2123	21.9	377	25.3	1733
Yes	75.4	4265	78.1	924	74.7	3309
Religion of household						
Muslim	58.9	2638	57.8	523	58.9	2093
Catholic	25.7	1135	21.7	185	26.7	945
Protestant	7.2	287	8.7	57	7.0	229
Traditional or other	8.2	223	11.8	61	7.5	162
Wealth tertile						
Poorest	34.2	1183	30.4	220	34.9	947
Middle wealth	31.8	1318	30.8	256	31.9	1049
Wealthiest	34.0	3887	38.8	825	33.2	3046
Residence						
Rural	77.8	2615	76.4	523	77.9	2063
Urban	22.2	3773	23.6	778	22.1	2979
Parity						
0	23.2	1875	19.7	319	24.1	1546
1-2	24.7	1729	24.9	349	24.7	1369
3-4	21.8	1409	23.9	323	21.3	1075
5+	30.3	1372	31.5	309	29.9	1052
Currently using contraception						
No	67.7	3998	71.0	882	66.7	3081
Yes	32.3	2390	29.0	419	33.3	1961
Currently using LARC						
No	86.1	5490	87.0	1130	85.8	4319
Yes	13.9	898	13.0	171	14.2	723
Induced abortion incidence	4.3	42	4.9	9	4.2	33
Total	100.0	6388	100.0	1301	100.0	5042

*Estimates weighted; bold indicates p-value for design-based F-test comparing respondents with 0 to 1+ friends less than 0.05
