# Supplementary material for: Induced abortion incidence and safety in Burkina Faso in 2020: Results from a population-based survey using direct and social network-based estimation approaches
Source: PLoS One. 2022 Nov 30;17(11):e0278168. doi: 10.1371/journal.pone.0278168 (PMC9710743; doi:10.1371/journal.pone.0278168)
Supplement: S2 Table — (PDF) [file pone.0278168.s002.pdf]

S2 Table. Induced abortion incidence (per 1,000) among female respondents aged 15 to 49 and their closest female friends aged 15 to 49 in Burkina Faso by background characteristics*

	Respondent			Adjusted friend**		
	Rate	95% CI		Rate	95% CI	
Age						
15-19	7.0	3.0	16.4	**30.6**	17.0	44.2
20-29	4.6	2.3	8.9	**27.9**	16.8	39.0
30-39	3.4	1.3	8.8	**19.2**	9.6	28.8
40-49	0.7	0.2	2.8	**9.9**	1.8	18.0
Education						
Never	1.6	0.0	3.2	**19.3**	11.3	27.2
Primary	3.4	1.2	5.7	**19.2**	9.3	29.0
Secondary	10.2	4.2	16.1	**33.6**	18.0	49.3
Tertiary	10.4	0.0	22.4	**30.0**	8.2	51.8
Currently married						
No	7.2	2.4	12.0	**35.8**	23.1	48.4
Yes	3.0	1.2	4.8	**18.4**	11.1	25.8
Wealth tertile						
Poorest	0.9	0.0	2.3	--	--	--
Middle wealth	3.9	0.5	7.2	--	--	--
Wealthiest	7.3	3.6	11.0	--	--	--
Residence						
Rural	3.4	1.3	5.6	**20.7**	11.5	29.8
Urban	6.2	2.8	9.5	**29.4**	21.4	37.4
Parity						
0	8.2	2.8	13.6	**34.8**	21.7	47.9
1+	2.8	1.1	4.4	**18.9**	11.5	26.4
Total	4.0	2.2	5.9	**22.9**	15.8	30.0

*Estimates weighted, Ns unweighted; bold indicates confidence intervals do not cross and are presumed to be significantly different.

**Estimates include respondent characteristics in place of "missing" friends; post-stratification weights applied
