# Supplementary material for: Induced abortion incidence and safety in Burkina Faso in 2020: Results from a population-based survey using direct and social network-based estimation approaches
Source: PLoS One. 2022 Nov 30;17(11):e0278168. doi: 10.1371/journal.pone.0278168 (PMC9710743; doi:10.1371/journal.pone.0278168)
Supplement: S3 Table — (PDF) [file pone.0278168.s003.pdf]

S3 Table. Percent of induced abortions that were unsafe among female respondents aged 15 to 49 and their closest female friends aged 15 to 49 in Burkina Faso by background characteristics*

	Respondent		Adjusted friend**	
	%	N	%	N
Age				
15-19	*80.1*	*12*	91.2	60
20-29	*80.8*	*54*	94.1	121
30-39	*97.1*	*38*	97.4	69
40-49	*100.0*	*17*	100.0	37
Education				
Never	94.4	44	**99.6**	**105**
Primary	90.9	27	**93.7**	**47**
Secondary	85.0	40	**91.3**	**123**
Tertiary	69.9	10	**67.3**	**13**
Currently married				
No	88.2	39	94.7	106
Yes	90.4	82	95.4	182
Wealth tertile				
Poorest	90.4	16	**--**	**--**
Middle wealth	92.3	26	**--**	**--**
Wealthiest	88.1	79	**--**	**--**
Residence				
Rural	93.0	49	**98.7**	**119**
Urban	83.8	72	**86.6**	**169**
Parity				
0	**76.4**	**26**	93.3	115
1+	**93.2**	**95**	96.0	173
Total	89.9	121	95.2	288

*Estimates weighted, Ns unweighted; bold indicates p<0.05, italics indicate p<0.10 from design-based F-test

**Adjusted friend data includes respondent abortion details for respondents who reported having no friends
